# Mitochondrial genomes and phylogenetic relationships of *Lates japonicus*, *Lates niloticus*, and *Psammoperca waigiensis* (Perciformes: Latidae)

**DOI:** 10.1080/23802359.2017.1285206

**Published:** 2017-02-06

**Authors:** Han Ming Gan, Hiroshi Takahashi, Michael P. Hammer, Mun Hua Tan, Yin Peng Lee, Jasmyn M. Voss, Christopher M. Austin

**Affiliations:** a Genomics Facility, Tropical Medicine and Biology Platform, Monash University Malaysia, Petaling Jaya, Malaysia;; b School of Science, Monash University Malaysia, Petaling Jaya, Malaysia;; c Department of Applied Aquabiology, National Fisheries University, Shimonoseki, Japan;; d Museum and Art Gallery of the Northern Territory, Darwin, Australia;; e School of Life and Environmental Sciences, Deakin University, Burwood, Australia

**Keywords:** Latidae, MiSeq, maximum likelihood

## Abstract

The complete mitochondrial genomes of four fish species of the commercially important family Latidae were sequenced using the Illumina MiSeq, thereby significantly increasing the mitogenomic resources for the family. Whole mitogenome-based phylogenetic analysis supports the monophyly of the genus *Lates* and more generally the family Latidae. The mitogenome sequences from this study will be useful for future assessments of the diversity within and between *Lates* species and studies of phylogenetic relationships within the diverse and taxonomically challenging perciform fishes.

The family Latidae currently contains 13 extant perch-like fishes from three genera, *Hypopterus* Gill, 1861, *Lates* Cuvier, 1828 and *Psammoperca* Richardson, 1848, which are found in a range of freshwater, brackish, and marine environments across Asia, Africa, and the Western Pacific and Indian oceans (Eschmeyer et al. 2016; Nelson et al. 2016). Despite low diversity, the family contains a number of commercially important species including the Asian sea bass or barramundi, *Lates calcarifer* and the Nile Perch, *L. niloticus*.

*Psammoperca waigiensis* or the Waigieu sea perch is the only member of the genus *Psammoperca* (Froese & Pauly 2016) and ranges widely across the Indo-West Pacific, from the Bay of Bengal to Japan to the northern and western coasts of Australia (Randall et al. 1990). In contrast, *L. japonicus* or akame or the Japanese lates has a relatively restricted range in the north western Pacific around Japan (Masuda et al. 1984). It is morphologically similar to both *P. waigiensis* and *L. calcarifer*, and was only declared a distinct species in 1984 (Katayama & Yasuhiko 1984). Unlike most of the other members of the family above, *L. niloticus* or the Nile perch is a purely freshwater species from north Africa that is of both economic importance and conservation concern as it has been introduced to several African lakes including the biological diverse Lake Victoria (Pringle 2005).

With the exception of *L. calcarifer* (Lin et al. 2006; Vij et al. 2014), genomic resources for the family Latidae are still fairly limited. In addition, from an evolutionary and taxonomic perspective, the status and relationships of the family to other Perciform groups is uncertain or unresolved (Vij et al. 2014; Harrington et al. 2016).

The sample of *L. japonicus* (Shi04) used for this study was collected from Shiomi River, Miyazaki Prefecture, Japan as described elsewhere (Takahashi et al. 2015). The sample of *P. waigiensis* was collected from Vesteys Beach, Darwin, in the Northern Territory, Australia (12.436° S, 130.829° E) with tissue and matched voucher specimen held at the Museum and Art Gallery of the Northern Territory (catalogue number: S.16708-010). The samples of the commercially important *L. calcarifer* and *L. niloticus* were obtained as muscle (fillets) from fish markets in Darwin, Australia sourced from the Northern Territory and Lake Victoria (imported), respectively.

Genomic DNA (gDNA) was extracted from approximately 50 mg fin clip or muscle tissue using the EZDNA Tissue extraction kit (OmegaBioTek, Norcross, GA). Approximately 100–200 ng of gDNA was sheared to 500 bp using a M220 focused-ultrasonicator (Covaris, Woburn, MA), prepped using NEBNext Ultra DNA library prep kit and subsequently sequenced on the MiSeq desktop sequencer (Illumina, San Diego, CA) located at the Monash University Malaysia Genomics Facility. Mitogenome assembly was performed using MITObim (Hahn et al. 2013). For *L. niloticus*, *L. japonicas*, and *P. waigiensis*, the *COX1* gene fragment for each species was used as the bait for iterative mapping assembly while for the *L. calcarifer* sample, the complete mitogenome already available for the species (GenBank: DQ010541) (Lin et al. 2006) was used instead. Each mitogenome was recircularized based on the presence of flanking repeat sequence as previously described (Gan et al. 2014) and annotated using MitoAnnotator (Iwasaki et al. 2013). For the construction of maximum-likelihood tree, 13 protein-coding genes and 2 ribosomal RNA genes were extracted, individually aligned (Abascal et al. 2010; Katoh & Standley 2014) and concatenated to form a super-alignment. The best-fit partitioning scheme was calculated using ModelFinder (Nguyen et al. 2015). Based on the optimum partitioning scheme, a maximum-likelihood (ML) tree was constructed using IQ-TREE with the 1000 ultrafast bootstrap approximation option to assess branch supports (Nguyen et al. 2015). The consensus tree was visualized and graphically edited using FigTree v 1.4.2 (http://tree.bio.ed.ac.uk/software/figtree/).

Partial genome sequencing successfully recovered the complete mitogenomes of all fish species obtained for this study, increasing the number of complete mitogenome for species of the family Latidae from one species (*L. calcarifer*) to a total of four. The latid mitogenomes have the typical structure organization of a fish mitogenome and consist of the typical 13 protein-coding genes, 22 transfer RNA genes and 2 ribosomal subunit genes, and a non-coding putative control region.

The mitogenome length and AT content ranges from 16,510 to 16,684 bp and 53.19 to 55.27%, respectively. The recovered mitogenomes exhibit high nucleotide similarity (> 95%) to the partial mitochondrial genes of members of the same species available on NCBI, providing support to their species designation based on morphology criteria or labelling as fish products. For example, our sample of *L. niloticus* was a fillet obtained from a fish market, but it had a 99% sequence similarity to the partial *COX1* gene of *L. niloticus* isolate FLN210 (GenBank: KJ443713), which was sampled in Egypt, the type locality for *L. niloticus* confirming the sample was true to label.

At the whole mitogenome level, *P. waigiensis* is the most divergent member of the Latidae sequenced with an average pairwise nucleotide identity of 79.8% (79.4–80.0%) to members of the genus *Lates*. The sample of *L. calcarifer* sequenced in this study is more similar to that of the Australian *L. calcarifer* isolate P12C03 (99.6%) at the *COX1* gene compared to that of Asian *L. calcarifer* (98.6%), which is consistent with its stated origin from near Darwin, Australia. Within the genus *Lates* our sample of *L. calcarifer* exhibits a similar pair-wise nucleotide identity (whole mitogenome alignment) to *L. japonicus* and *L. niloticus* (86.7% and 86.2%, respectively).

ModelFinder identified four partitions as the best-fit partitioning scheme for the 13 PCG and 2 rRNA genes utilized for phylogenetics inference as follows: Partition1 = 12S and 16S; Partition2= *ATP6*, *CYTB*, *COX3*, *ND3*, *COX1*, *COX2*; Partition3= *ATP8*, *ND3*, *ND3 ND4L*, *ND2*; Partition4= *ND6*). Phylogenetic analysis using maximum likelihood, based on the final partitioned nucleotide alignment consisting of 14,270 DNA characters, supports the monophyly of the genus *Lates* and to a certain extent the monophyly of the families Latidae, Carangidae, and Xiphiidae (Figure 1). However, within the Latidae a sister grouping between *L. japonicus* and *L. calcarifer* was only weakly supported (ultrafast bootstrap support =56%) and the relationships among all three species of *Lates* should be considered pending further taxon and gene sampling. In general, the evolutionary relationships among the different families included in the study are poorly supported and may similarly benefit from a more extensive taxon and gene sampling, which is beyond the scope of this study.

**Figure 1.**
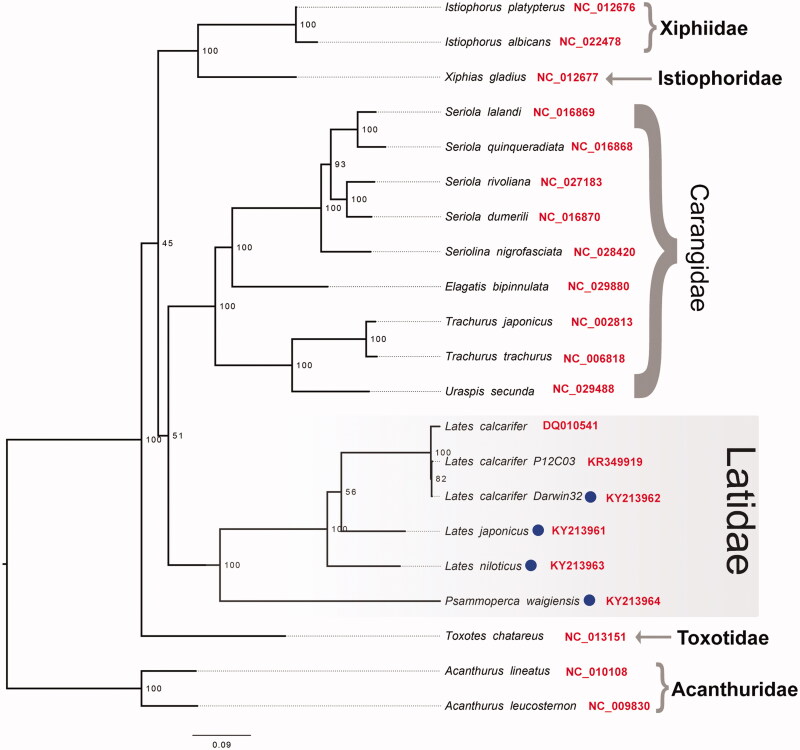
Phylogenetic tree depicting the evolutionary relationships within the order, inferred from maximum-likelihood estimation based on 13 mitochondrial protein-coding genes and two ribosomal RNA genes with best-fit partitioning scheme. Members from the family Acanthuridae were used as an outgroup and a selection of species from families thought to be related to the Latidae were included for comparative purposes. Circles next to tip labels indicate sequence reported in this study. NCBI accession numbers were provided next to tip labels (in red). Numbers at nodes indicate Ultrafast Bootstrap support and branch lengths indicate number of substitutions per site.

## References

[CIT0001] Abascal F, Zardoya R, Telford MJ. 2010 TranslatorX: multiple alignment of nucleotide sequences guided by amino acid translations. Nucleic Acids Res. 38:W7–13. 20435676 10.1093/nar/gkq291 PMC2896173

[CIT0002] Eschmeyer WN, Fricke R, Van Der Laan R. 2016 Catalog of fishes: Genera, Species, References [Internet]. [cited 2016 Nov 17]. Available from: http://researcharchive.calacademy.org/research/ichthyology/catalog/fishcatmain.asp

[CIT0003] Froese R, Pauly D. 2016. FishBase (version Aug 2016) In: Roskov Y, Abucay L, Orrell TM, Nicolson D, Flann C, Bailly N, Kirk P, Bourgoin T, Dewalt RE, DECOCK W, de Wever A, editors. Species 2000 & ITIS catalogue of life. Naturalis, Leiden, the Netherlands: Species 2000.

[CIT0004] Gan HM, Schultz MB, Austin CM. 2014 Integrated shotgun sequencing and bioinformatics pipeline allows ultra-fast mitogenome recovery and confirms substantial gene rearrangements in Australian freshwater crayfishes. BMC Evol Biol. 14:19. 24484414 10.1186/1471-2148-14-19 PMC3915555

[CIT0017] Hahn C, Bachmann L, Chevreux B. 2013 Reconstructing mitochondrial genomes directly from genomic next-generation sequencing reads–a baiting and iterative mapping approach. Nucleic Acids Res. 41:e129. 23661685 10.1093/nar/gkt371 PMC3711436

[CIT0005] Harrington RC, Faircloth BC, Eytan RI, Smith WL, Near TJ, Alfaro ME, Friedman M. 2016 Phylogenomic analysis of carangimorph fishes reveals flatfish asymmetry arose in a blink of the evolutionary eye. BMC Evol Biol. 16:224. 27769164 10.1186/s12862-016-0786-x PMC5073739

[CIT0006] Iwasaki W, Fukunaga T, Isagozawa R, Yamada K, Maeda Y, Satoh TP, Sado T, Mabuchi K, Takeshima H, Miya M, Nishida M. 2013 MitoFish and MitoAnnotator: a mitochondrial genome database of fish with an accurate and automatic annotation pipeline. Mol Biol Evol. 30:2531–2540. 23955518 10.1093/molbev/mst141 PMC3808866

[CIT0007] Katayama M, Yasuhiko T. 1984 Lates japonicus, a New Centropomid Fish from Japan. JpnJ Ichthyol. 30:361–367.

[CIT0008] Katoh K, Standley DM. 2014 MAFFT: iterative refinement and additional methods. Methods Mol Biol. 1079:131–146. 24170399 10.1007/978-1-62703-646-7_8

[CIT0009] Lin G, Lo LC, Zhu ZY, Feng F, Chou R, Yue GH. 2006 The complete mitochondrial genome sequence and characterization of single-nucleotide polymorphisms in the control region of the Asian seabass (Lates calcarifer). Mar Biotechnol (NY). 8:71–79. 16228120 10.1007/s10126-005-5051-z PMC4273291

[CIT0010] Masuda H, Amaoka K, Araga C, Uyeno T. 1984 The fishes of the Japanese Archipelago. Tokyo, Japan: Tokai University Press.

[CIT0011] Nelson JS, Grande TC, Wilson MVH. 2016 Fishes of the world. 5th ed Hoboken (NY): John Wiley & Sons.

[CIT0012] Nguyen LT, Schmidt HA, Von Haeseler A, Minh BQ. 2015 IQ-TREE: a fast and effective stochastic algorithm for estimating maximum-likelihood phylogenies. Mol Biol Evol. 32:268–274. 25371430 10.1093/molbev/msu300 PMC4271533

[CIT0013] Pringle RM. 2005 The Origins of the Nile Perch in Lake Victoria. BioScience. 55:780–787.

[CIT0014] Randall JE, Allen GR, Steene RC. 1990 Fishes of the Great Barrier Reef and Coral Sea. Honolulu, Hawaii: University of Hawaii Press.

[CIT0015] Takahashi H, Takeshita N, Tanoue H, Ueda S, Takeshima H, Komatsu T, Kinoshita I, Nishida M. 2015 Severely depleted genetic diversity and population structure of a large predatory marine fish (Lates japonicus) endemic to Japan. Conserv Genet. 16:1155–1165.

[CIT0016] Vij S, Purushothaman K, Gopikrishna G, Lau D, Saju JM, Shamsudheen KV, Kumar KV, Basheer VS, Gopalakrishnan A, Hossain MS, et al 2014 Barcoding of Asian seabass across its geographic range provides evidence for its bifurcation into two distinct species. Front Mar Sci. [Internet]. Available from: http://journal.frontiersin.org/article/10.3389/fmars.2014.00030/full

